# Functional centrality as a predictor of shifts in metabolic flux states

**DOI:** 10.1186/s13104-016-2117-0

**Published:** 2016-06-21

**Authors:** Max Sajitz-Hermstein, Zoran Nikoloski

**Affiliations:** Systems Biology and Mathematical Modeling Group, Max Planck Institute of Molecular Plant Physiology, Am Mühlenweg 1, 14476 Potsdam, Germany

**Keywords:** Functional centrality, Metabolic network, Flux balance analysis, Elementary flux mode, Reaction stoichiometry, Structural modeling, Metabolic control, Metabolic flux, Cooperative game theory, Shapley value

## Abstract

**Background:**

The flux phenotype describes the entirety of biochemical conversions in a cell, which renders it a key characteristic of metabolic function. To quantify the functional relevance of individual biochemical reactions, functional centrality has been introduced based on cooperative game theory and structural modeling. It was shown to be capable to determine metabolic control properties utilizing only structural information. Here, we demonstrate the capability of functional centrality to predict changes in the flux phenotype.

**Results:**

We use functional centrality to successfully predict changes of metabolic flux triggered by switches in the environment. The predictions via functional centrality improve upon predictions using control-effective fluxes, another measure aiming at capturing metabolic control using structural information.

**Conclusions:**

The predictions of flux changes via functional centrality corroborate the capability of the measure to gain a mechanistic understanding of metabolic control from the structure of metabolic networks.

## Background

The metabolic state of a cellular system is characterized by the fluxes of the underlying biochemical reactions and the concentrations of the involved metabolites [1]. In the analysis of metabolism, the flux phenotype is one of the most important cellular observables as it directly relates to metabolic functionality, i.e., the conversion of individual biochemical compounds [2], which also naturally accounts for changes in metabolic state.

Alterations in the flux phenotype are triggered by internal or external shifts and imply rerouting of metabolic flux, for example from respiration towards fermentation in facultative aerobic bacteria [3], which is the result of metabolic control [1]. Several constraint-based approaches were proposed to predict flux phenotypes in unicellular organisms resulting from genetic interventions by minimizing their effect on flux distributions [4, 5]. Nevertheless, elucidating the principles of how large-scale metabolic systems achieve efficient metabolic rerouting remains one of the key challenges in developing a mechanistic understanding of metabolic control, in accordance with the general case of controlling complex networks [6]. Here, we demonstrate the ability of our recently introduced measure of *functional centrality* to predict changes in experimentally determined metabolic fluxes based solely on the structure of the metabolic network captured by its stoichiometric matrix.

Functional centrality combines cooperative game theory, in particular a modified version of the Shapley value for arbitrary restricted games [7], with flux balance analysis (FBA) [8] to determine the functional relevance of biochemical reactions [1, 9]. It quantifies the contribution of individual biochemical reactions to metabolic functionality, e.g., to biomass production, based on the structure of a metabolic network together with constraints on internal and exchange fluxes, which mimic internal and external conditions.

**Fig. 1:**
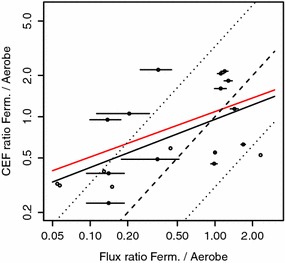
Prediction of flux changes by control-effective fluxes. Calculated ratios between flux during growth on glucose (GLC) under conditions of aerobic respiration and fermentative conditions based on control-effective fluxes versus experimentally determined flux changes (double-logarithmic plot). *Lines* indicate $95\,\%$ confidence intervals for experimental data (*horizontal lines*), linear regression (*solid line*), perfect match (*dashed line*) and twofold deviation (*dotted line*). Some data points are shown without error bars, because the corresponding error intervals exhibit partially negative values; linear regression fit only for the data points with strictly positive error intervals is shown in *red* ($R^{2}=0.1934$)

**Fig. 2:**
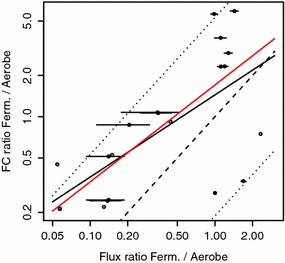
Prediction of flux changes by functional centralities. Calculated ratios between flux during growth on glucose (GLC) under conditions of aerobic respiration and fermentative conditions based on functional centralities versus experimentally determined flux changes (double-logarithmic plot). Lines indicate $95\,\%$ confidence intervals for experimental data (*horizontal lines*), linear regression (*solid line*), perfect match (*dashed line*) and twofold deviation (*dotted line*). Some data points are shown without error bars, because the corresponding error intervals exhibit partially negative values; linear regression fit only for the data points with strictly positive error intervals is shown in *red* ($R^{2}=0.3489$)

In our previous study we examined the relationship of functional relevance and transcriptional readout [1], whereby we examined four measures based on structural modeling and associated with metabolic control. Our comparative analysis showed that control-effective fluxes [10] were superior in predicting changes of gene expression. However, gene expression may not directly be manifested in changes of flux; a more suitable comparison necessitates the usage of changes in fluxes inferred based on labeling data [11]. In this brief research note, we demonstrate that functional centrality performs better than control-effective fluxes in predicting changes in metabolic flux in the central carbon metabolism of *Escherichia coli*.

## Results

We examine the capability to predict flux changes using functional centralities as well as control-effective fluxes, which were found to be the two best-performing contending predictors of transcriptional change in a recent study of structural control of metabolism [1]. To this end, we analyze the relationship of flux changes in the central carbon metabolism of *E. coli* resulting from a shift between aerobic and fermentative conditions and the corresponding changes of the two investigated measures. The flux data were obtained via stationary ^13^C labeling experiments and metabolic flux analysis by Fischer et al. [12].

In accordance with the definition of control-effective fluxes [10], we utilize the mean of normalized functional centralities obtained for the metabolic functions of ATP and biomass production, which are the two dominant metabolic functions shaping the flux phenotype [13]. The control-effective fluxes and the mean functional centralities are calculated for aerobic as well as for fermentative conditions using the metabolic network of *E. coli*’s central carbon metabolism provided by Schuetz et al. [13]. For both measures, we calculate the ratios of values obtained for the two conditions and use them as predictors of ratios of the corresponding fluxes. Estimates of functional centralities are used, because exact computation is computationally intractable for large metabolic network. We demonstrated that robust estimates of functional centralities can be obtained via Monte Carlo sampling utilizing the set of elementary flux modes in medium-size metabolic networks [1].

**Table 1:** Association of flux changes and their predictions

	FC	p value	CEF	p value
Kendall’s $\tau$	0.63	${<}3\cdot 10^{-5}$	0.45	${<}3\cdot 10^{-3}$
Pearson’s *r*	0.50	0.016	0.39	0.065
Spearman’s $\rho$	0.74	${<}5\cdot 10^{-5}$	0.67	${<}5\cdot 10^{-4}$

Changes are caused by switching from aerobic to fermentative conditions; predictors are changes of functional centralities (FC) and of control-effective fluxes (CEF), respectively

The ability of control-effective fluxes and functional centrality to predict the observed flux changes (23 data points) is assessed by employing two statistical procedures. In the first, we fit a regression model. Inspection of the dataset indicates a non-linear relationship in the case of both measures. We utilize logarithmic transformation of the predictor’s and of the regressor’s values, respectively, to discount non-linearity and to render the distribution of the predicted quantity closer to normal. A linear regression model is fitted to the double-log-transformed data (residuals do not show significant deviations from a normal distribution). The slope of the linear regression line then indicates the relative (percental) change of flux resulting from a relative (percental) change of the predictor. The goodness of fit is determined by the coefficient of determination

1 $$\begin{aligned} R^{2}=1-\frac{\sum _{i}R_{i}^{2}}{\sum _{i}(y_{i}-\bar{y})^{2}}, \end{aligned}$$

whereby $y_{i}$ and $y_{i}^{*}$ denote the *i*th (log-transformed) data point and the (log-transformed) predicted value, respectively; $R_{i}=y_{i}-y_{i}^{*}$ denotes the *i*th residual and $\bar{y}$ the mean of data points. In the second, we use Kendall’s rank correlation coefficient. We find that functional centrality improves prediction of metabolic fluxes in comparison to control-effective fluxes (see Figs. 1, 2). While control-effective fluxes can only explain $35.1\,\%$ of the variance of measured fluxes, functional centrality is capable to explain $44.21\,\%$ of the variance. The association found by utilizing Kendall’s rank correlation coefficient is even more pronounced as shown in Table 1, which also provides values of Pearson’s and Spearman’s correlation coefficient.

## Conclusions

Functional centrality, like control-effective fluxes, does not require any further information besides reaction stoichiometry, characterization of exchange fluxes and the choice of a metabolic function. Here we demonstrated that functional centrality enables prediction of changes in metabolic flux with improved accuracy, exceeding the results of control-effective fluxes. The functional centrality measure is based on the rich axiomatic framework of power indices in cooperative games, and is suitable for the analysis of structural control of metabolic networks, as supported by the analyzed data. Our results provide further indication that concepts from cooperative game theory can help in obtaining a better understanding of control in metabolic networks.

## Methods

### Flux balance analysis

Flux balance analysis is a structural modeling framework enabling prediction of steady-state metabolic flux [8]. It relies on the assumption that metabolism is governed by an optimization principle with respect to some cellular objective. Usually, the objective is expressed as a linear combination of individual fluxes $f_{i}$ ($i\in \mathcal {N},$ with $\mathcal {N}$ denoting the set of reactions forming the metabolic network). Optimization can then be formulated in terms of a linear programming problem:

2 $$\begin{aligned} \text {min(max)}z=\sum _{i}c_{i}f_{i},\text { s.t.}\nonumber \\ \mathbf {S}\cdot \mathbf {f}=\mathbf {0},\nonumber \\ f_{min}(i)\le f_{i}\le f_{max}(i), \end{aligned}$$

with *z* representing the objective to be optimized, $\mathbf {c}$ being a vector of coefficients quantifying the contribution of each flux to this objective, and matrix $\mathbf {S}$ capturing reaction stoichiometries. The bounds $f_{min}(i)$ and $f_{max}(i)$, denote minimum and maximum values of the fluxes and, therefore, determine reaction reversibility.

### Functional centrality

Functional centrality quantifies the contribution of individual reactions to a metabolic function, e.g., biomass production, by utilizing a formulation of the Shapley value for restricted cooperative games [9]. The metabolic function of interest is formulated in terms of a linear objective function accessible via FBA. We provide briefly the definition of functional centrality, an extensive derivation and an algorithm for estimation of functional centrality in large networks is provided by Sajitz-Hermstein and Nikoloski [1, 9].

The optimal value of the objective function *v* in a subnetwork formed by the reaction set *S* is determined by FBA. Let $G_{\mathcal {S}}=(\mathcal {S},\mathcal {A})$ be a directed graph. The set of nodes $\mathcal {S}$ encompasses all subsets $S\subseteq \mathcal {N}$ which correspond to functional subnetworks, i.e., exhibiting positive outcome of FBA, and the empty set $\emptyset$. The set of arcs $\mathcal {A}$ consists of all $(S,S')$ with $S\subsetneq S'$ for which it holds that there exists no $S''\in \mathcal {S}$ with $S\subsetneq S''\subsetneq S'$. In $G_{\mathcal {S}}$, every path from the empty set to the set $\mathcal {N}$ represents one possibility to add reactions successively in such a way that the corresponding subnetworks belong to the family of functional subnetworks. These paths are called *maximal chains*. The calculation of functional centrality is as follows:

Let $W=(S_{0},S_{1},\ldots ,S_{l(W)})$ with $S_{0}=\emptyset$ and $S_{l(W)}=\mathcal {N}$ be a maximal chain for inclusion in $\mathcal {S}$, implying $S_{0}\subsetneq S_{1}\subsetneq \cdots \subsetneq S_{l(W)}.$ Then, the contribution of reaction *i* in maximal chain *W* is given by

3 $$\begin{aligned} \psi _{W,i}(v)=\sum _{j=1}^{l(W)}\frac{v(S_{j})-v(S_{j-1})}{|S_{j}|-|S_{j-1}|}\cdot \chi (S_{j}\setminus S_{j-1},i)\quad \text { for }i\in \mathcal {N}, \end{aligned}$$

4 $$\begin{aligned} \chi (S,i)= {\left\{ \begin{array}{ll} 1 &{} \quad \text {if }\, i\in S\\ 0 &{} \quad \text {otherwise}, \end{array}\right. } \end{aligned}$$

with *l*(*W*) being the length of the maximal chain *W* and $|\cdot |$ denoting the cardinality of a set. Let the set of all maximal chains be denoted by $\mathcal {W}.$ The functional centrality $\Phi _{i}(v)$ of reaction *i* with respect to the objective function *v* is

5 $$\begin{aligned} \phi _{i}(v)=\sum _{W\in \mathcal {W}}\frac{1}{|\mathcal {W}|}\psi _{W,i}(v). \end{aligned}$$

### Control-effective fluxes

Control-effective fluxes are defined by *efficiencies*$\varepsilon _{i}$ of the individual elementary flux modes (EFM) $e_{i}$ with respect to a substrate $S_{k}$ and the production of biomass ($\mu$) and ATP (EFMs are normalized by substrate uptake in advance) [10]:

6 $$\begin{aligned} \varepsilon _{i}(S_{k},\mu ) = {} \frac{e_{i}^{\mu }}{\sum _{l}|e_{i}^{l}|},\end{aligned}$$

7 $$\begin{aligned} \varepsilon _{i}(S_{k},\mathrm {ATP}) = {} \frac{e_{i}^{\mathrm {ATP}}}{\sum _{l}|e_{i}^{l}|}, \end{aligned}$$

whereby $e_{i}^{l}$ denotes the flux through reaction *l* in the EFM $e_{i}.$ The control-effective fluxes are then given by

8 $$\begin{aligned} C_{l}(S_{k}) &=\frac{1}{Y_{\mu /S_{k}}^{\mathrm {max}}}\cdot \frac{\sum _{i}\varepsilon _{i}(S_{k},\mu )\cdot |e_{i}^{l}|}{\sum _{i}\varepsilon _{i}(S_{k},\mu )}+\frac{1}{Y_{\mathrm {ATP}/S_{k}}^{\mathrm {max}}} \\ & \quad \cdot \frac{\sum _{i}\varepsilon _{i}(S_{k},\mathrm {ATP})\cdot |e_{i}^{l}|}{\sum _{i}\varepsilon _{i}(S_{k},\mathrm {ATP})}, \end{aligned}$$

with $Y_{(\mu /ATP)/S_{k}}^{\mathrm {max}}$ being the maximum yield of biomass or ATP production, respectively, for substrate $S_{k}$.

### Model

We utilize a model of central carbon metabolism of *E. coli* originally published by Schuetz et al. [13]. Import of acetate and ethanol is disabled in our study. The sum of glucose import by the reactions *mglABC* and *ptsGHI* is constrained from above arbitrarily by one. Isozymes in the model were deleted. We examine two environmental conditions: (i) *aerobic respiration* and (ii), *fermentation* (no oxygen and no nitrate import). The utilized objective functions are the fluxes through the reactions: (i) *maint* (ATP production) and (ii), *biomass* (biomass production).

## Abbreviations

FBA: flux balance analysis
EFM: elementary flux modes
